# Neural decoding of music from the EEG

**DOI:** 10.1038/s41598-022-27361-x

**Published:** 2023-01-12

**Authors:** Ian Daly

**Affiliations:** Brain-Computer Interfacing and Neural Engineering Lab, Department of Computer Science and Electronic Engineering, University of Essex, Colchester, CO4 3SQ UK

**Keywords:** Machine learning, Design, synthesis and processing, Imaging techniques, Cognitive neuroscience, Computational neuroscience

## Abstract

Neural decoding models can be used to decode neural representations of visual, acoustic, or semantic information. Recent studies have demonstrated neural decoders that are able to decode accoustic information from a variety of neural signal types including electrocortiography (ECoG) and the electroencephalogram (EEG). In this study we explore how functional magnetic resonance imaging (fMRI) can be combined with EEG to develop an accoustic decoder. Specifically, we first used a joint EEG-fMRI paradigm to record brain activity while participants listened to music. We then used fMRI-informed EEG source localisation and a bi-directional long-term short term deep learning network to first extract neural information from the EEG related to music listening and then to decode and reconstruct the individual pieces of music an individual was listening to. We further validated our decoding model by evaluating its performance on a separate dataset of EEG-only recordings. We were able to reconstruct music, via our fMRI-informed EEG source analysis approach, with a mean rank accuracy of 71.8% ($n~=~18$, $p~<~0.05$). Using only EEG data, without participant specific fMRI-informed source analysis, we were able to identify the music a participant was listening to with a mean rank accuracy of 59.2% ($n~=~19$, $p~<~0.05$). This demonstrates that our decoding model may use fMRI-informed source analysis to aid EEG based decoding and reconstruction of acoustic information from brain activity and makes a step towards building EEG-based neural decoders for other complex information domains such as other acoustic, visual, or semantic information.

## Introduction

Neural decoding models attempt to identify the current mental state of an individual from recordings of their neural activity^1^. In recent years, neural decoders have been developed to identify numerous different types of mental activity from many neuroimaging modalities. These decoders were first developed to decode visual^2,3^ and semantic^4–7^ information from the brain, while more recent examples of neural decoders have been developed to decode a diverse set of activities, including, but not limited to, affective states^8^, visual imagery during sleep^9^, and story meaning^10^.

Neural decoding models have been developed that make use of many different types of neuroimaging techniques including, but not limited to, functional magnetic resonance imaging (fMRI), electrocortiography (ECoG), electroencephalogram (EEG), and functional near infrared spectroscopy (fNIRS). Depending on the type of neuroimaging technique the neural decoder uses different types of mental processes may be decoded. For example, fMRI provides a recording of activity throughout the entire brain with a very high spatial resolution, allowing a neural decoder the ability to decode mental states involving sub-cortical brain regions^11^. However, this comes at the cost of poor time resolution, which prevents decoding of mental activity over very short time scales. Near the other end of the spatial–temporal resolution axis, EEG provides a much higher temporal resolution allowing decoding of mental activity over very short time scales, but this comes at the cost of much poorer spatial resolution, limiting accurate decoding of activity located in sub-cortical brain regions.

The majority of neural decoding models developed to date make use of the fMRI, while a smaller number of decoding models make use of the invasive neuroimaging technique ECoG. A few neural decoding models focused in the semantic and acoustic information domains make use of non-invasive neuroimaging techniques that are not fMRI, such as EEG or fNIRS^7^. These non-invasive neuroimaging modalities have significantly poorer spatial resolutions and are highly susceptible to noise contamination, which makes their use in decoding models considerably more challenging.

Broadly speaking decoding models have two different uses, reconstruction of the original stimulus or mental activity, and identification of the category of stimulus or mental imagery. Both uses have many potential applications, including neuroscientific and psychological research^12,13^, as well as potential uses in diagnostic medicine^14–16^.

Neural decoding models that are able to decode acoustic information in particular have numerous potential applications. One such exciting application is in the field of brain–computer interfacing (BCI)^17^. BCIs provide a communication channel directly between the brain and a computer device. However, their speed and accuracy is limited by the performance of the neural decoding models used within BCI systems, which is itself constrained by the use of non-invasive neuroimaging modalities, such as EEG, in BCI. Neural decoders that are able to accurately decode acoustic information from the EEG have numerous applications, including dramatically improving BCI system performance.

Some neural decoding models have been developed for decoding acoustic information. The vast majority of these decoding models developed to date use either fMRI or ECoG neuroimaging techniques^18^. For example, in work by Anumanchipalli et al. ECoG was used to attempt to decode speech from a group of five participants with error rates of 31% from a set of 50 words^19^. In another study by Hoefle et al. fMRI was used to attempt to decode the music a cohort of 6 participants heard with a mean accuracy of 76%^20^. On the other hand a relatively smaller, but growing, number of decoding models have also been developed to attempt to decode accoustic information from the EEG^21^. For example, recent work by Di Liberto et al. attempted to use decoding models to identify musical bars from the EEG^22^, while Tsekoura and Foka attempted to identify individual musical notes from the EEG with an average accuracy of 63.7%.

Several researchers have explored the use of EEG based accoustic decoders to identify the individual song a participant was listening to. For example, Marion et al. used a decoding model to identify individual songs participants listened to and imagined from their EEG^23^. The performance of these EEG based accoustic decoders for song identification varies considerably across studies. For example, many decoders achieve classification accuracies, for differentiating between 7 to 12 pieces of music, of between 23 and 50%^24–28^. However, a few studies report much higher song identification accuracies of between 80 and 88%^21,29,30^.

Other studies have attempted to reconstruct the music participants listened to from their EEG. For example, Di Liberto et al. attempted to identify the note onset times and the accoustic time series from the EEG and achieved accuracies of approximately 90–100% for note onset times and correlations of $r~=~0.6$ for reconstruction of the accoustic time series^31^. Others have attempted to reconstruct aspects of the accoustic signal with correlations between the reconstructed signal and the original audio varying between $r~=~0.2$ and $r~=~0.4$^32,33^.

Other researchers have begun the develop the closely related field of music information retrieval^28,34^, which aims to retrieve musical information from the EEG, with some initial successes^21,35^. However, there remains a need to explore other novel ways to identify music from EEG and, in general, there is a sparsity of decoding models that attempt to use multi-modal non-invasive cortical neuroimaging techniques such as combinations of EEG and fMRI.

To address this issue we build and validate a neural decoding model to both reconstruct and identify the music an individual is listening to from a combination of EEG and fMRI recordings of their brain activity. Music is a form of emotional communication and is also a complex acoustic signal that shares many temporal, spectral, and grammatical similarities with human speech. Thus, a neural decoding model that is able to reconstruct heard music from brain activity can form a reasonable step towards other forms of neural decoding models that have applications for aiding communication.

We adapt a method first developed for speech synthesis from invasively recorded ECoG signals^19^ to see if a similar approach can be used to decode music from non-invasive neural data. Specifically, we aim to recover the music an individual is listening to using fMRI-informed EEG source analysis.

We construct an fMRI-informed EEG source analysis approach to extract EEG features related to music listening. We then use a bi-directional long term short term (biLSTM) deep neural network to construct a neural decoding model that is able to reconstruct the music heard by a participant from their EEG. We use this neural decoding model to reconstruct heard music from a cohort of 18 participants and then evaluate the efficacy of our reconstructed music by using it to attempt to identify which piece of music (from a set of 36 different music pieces) each participant was listening to within each music listening epoch.

We further validate the efficacy of our neural decoding model by applying it to reconstruct and identify heard music in a second dataset containing just EEG (without fMRI) recorded from a further cohort of 19 participants.

## Results

### Participants

Our two datasets contain neuroimaging data recorded from participants as they listen to music. Our first dataset contains jointly recorded EEG and fMRI recorded from a cohort of 20 adult participants. Our second dataset contains just EEG recorded from a different cohort of 20 adult participants. During analysis of these datasets problems were found with 2 participants in the EEG-fMRI dataset and 1 participant in the EEG-only dataset. Specifically, artifacts and other minor numerical problems with some parts of the data for these participants prevented effective convergence of the biLSTM training process. Consequently, these participants were removed from the final analysis, leaving 18 participants in the EEG-fMRI dataset and 19 participants in the EEG-only dataset.

### fMRI results

**Figure 1:**
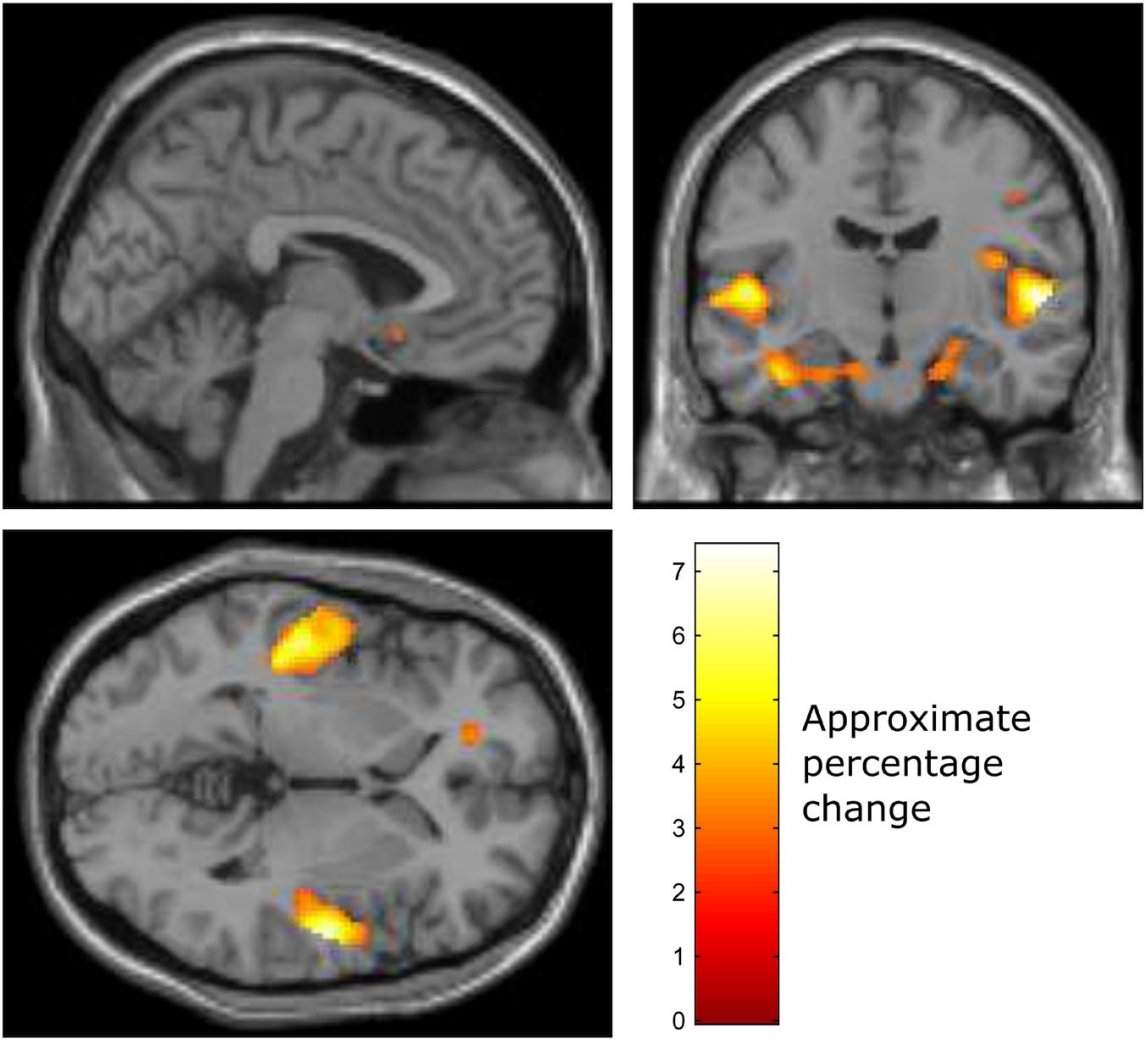
Voxels that significantly differ in activity between music listening and no music trials over all participants ($p< 0.001$, not corrected for multiple comparisons). The colour scale indicates approximate percentage change in BOLD activity. The voxel activities are averaged over all participants (2nd level analysis) and fitted to a standard T1-weighted image.

The mean set of voxels over all participants, in our EEG-fMRI dataset, that were found to significantly differ in BOLD activity levels between music listening and no music trials over all participants (2nd level analysis) is illustrated in Fig. 1. This figure provides a partial illustration of some of the significant clusters of voxels we identified. A complete list of the locations and sizes of the clusters of voxels are listed in Table 1.

**Table 1:** Local maxima of brain regions with significantly different levels of BOLD activity between music and no music trials (voxel size: $2~\times ~2~\times ~2$ mm).

Anatomical region	Voxels	Location (mm)	p	T-score
Right transverse temporal gyrus	614	54, − 12, 2	< 0.0001	7.38
Left transverse temporal gyrus	1132	− 50, − 20, 2	< 0.0001	6.62
Left hippocampus	121	− 22, − 26, − 16	< 0.0001	5.67
Right cerebellum exterior	55	16, − 38, − 10	< 0.0001	4.45
Left cerebellum exterior	34	− 18, − 40, − 18	< 0.0001	4.20
Left parahippocampal gyrus	33	− 8, − 4, − 22	< 0.0001	4.06
Right parahippocapal gyrus	67	32, − 26, − 22	< 0.0001	3.99

Locations are reported in the Montreal Neurological Institute (MNI) coordinate sytem.

A network of several brain regions exhibit activity that significantly differs between the music and no music trials. These brain regions include many parts of the auditory response brain network^36^, including the left and right auditory cortex (containing the left and right transverse temporal gyri) and the cerebellum, as well as the hippocampus, which is a part of the emotion response network that is known to be involved in emotional responses to music^37^.

**Figure 2:**
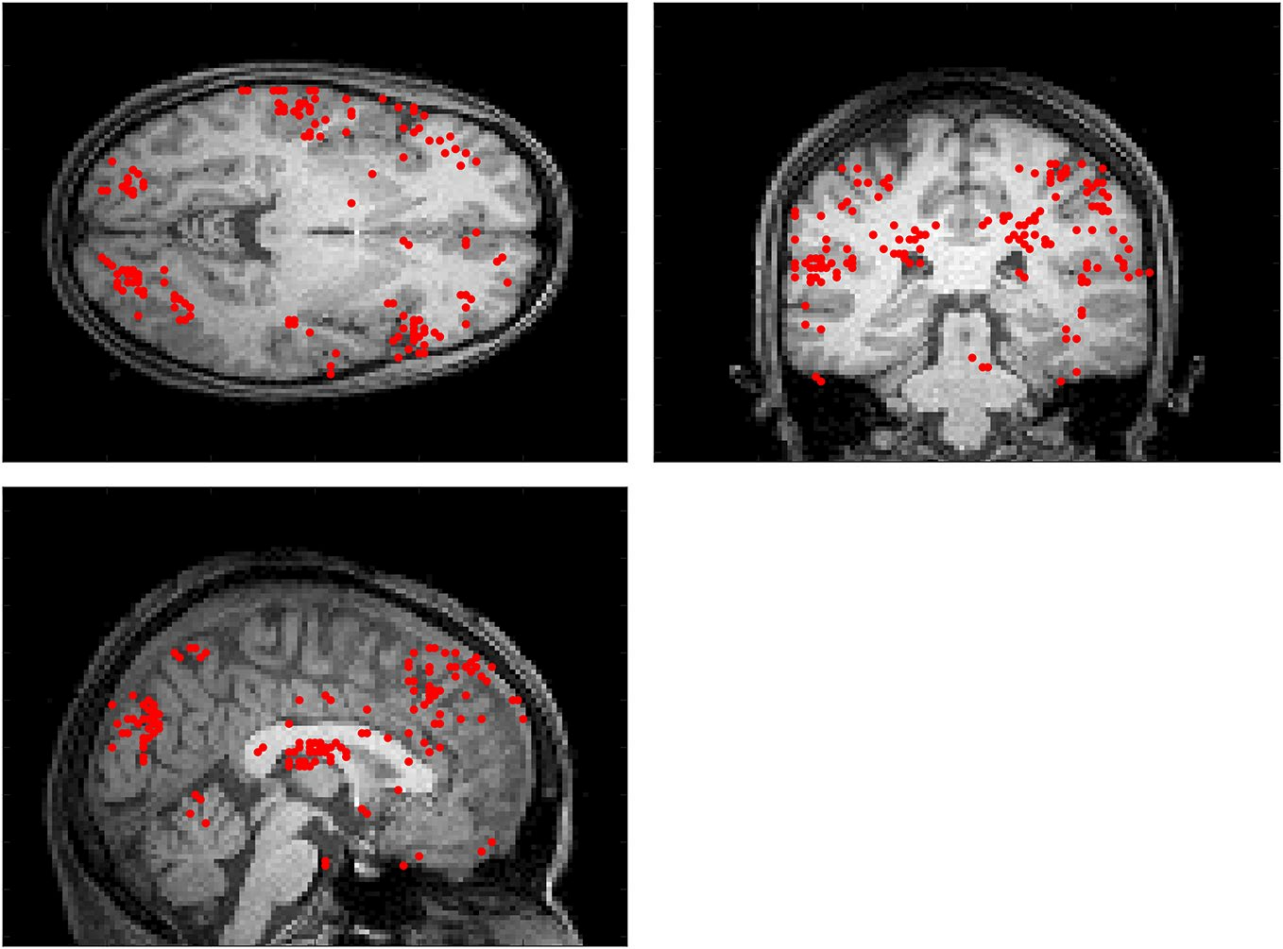
Locations of the 4 dipole locations chosen for each fold of the $3~\times ~3$ cross fold validation scheme for each participant and overlaid on a standard T1-weighted image projected on the axial, coronal, and sagittal planes.

The dipole locations used for EEG source analysis are chosen individually for each participant from the fMRI analysis results for that participant. Figure 2 illustrates the locations of the four most significant dipole locations chosen for each participant that are used in our fMRI-informed EEG source analysis.

The locations of the dipoles identified from our fMRI analysis and used for our fMRI-informed EEG source analysis are clustered in a few key brain regions, including the left and right temporal cortices and the paratiel cortex. These are similar to the brain regions identified via our GLM analysis of the fMRI recordings as differentiating music listening vs. no music trials. However, it is also important to note that there is considerable variation in voxel locations between participants, a finding which highlights the need to use individual fMRI-informed source analysis for each participant.

### Recovered music

**Figure 3:**
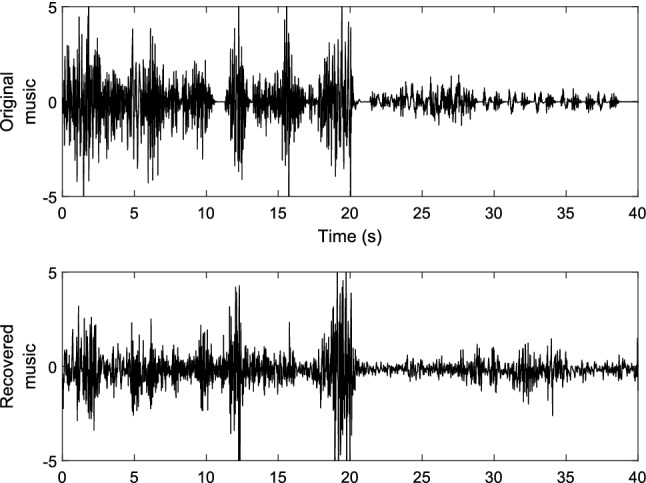
An example of the time series of the music played to the participants (top) and the music reconstructed via our fMRI-informed EEG source analysis and biLSTM decoder. The music is downsampled to the same sample rate as the EEG (1000 Hz) and the amplitudes have been z-scored.

An example of the time series of the amplitudes of the music recovered via our neural decoding model is illustrated in Fig. 3 along with the original music played to the participant. The amplitudes of both signals have been z-scored to aid visualisation.

The decoded music and the original music time series share some clear visual similarities in their amplitudes over time. In particular, changes in amplitude over time in the original music are clearly recovered effectively, including recovering the amplitude envelopes of several individual notes and a good approximation of the overall amplitude envelope of the piece of music. This suggests that our decoding approach is able to reconstruct at least some of the temporal and spectral content of the music from the EEG.

**Figure 4:**
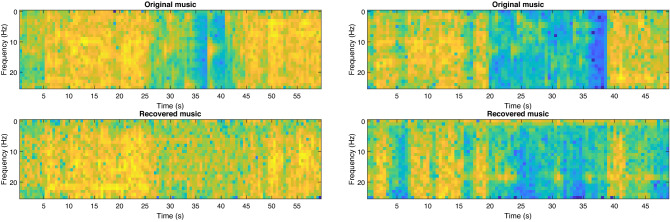
An example of the time-frequency spectrograms of the music played to the participants (top) and the music reconstructed via our fMRI-informed EEG source analysis and biLSTM decoder. The music is downsampled to the same sample rate as the EEG (1000 Hz) and then further downsampled for decoding to 100 Hz.

We also illustrate examples of the time-frequency spectrograms of the original and decoded music in Fig. 4. It can be seen that there are clear visual similarities between the original and decoded music at a wide range of different time and frequency locations.

**Figure 5:**
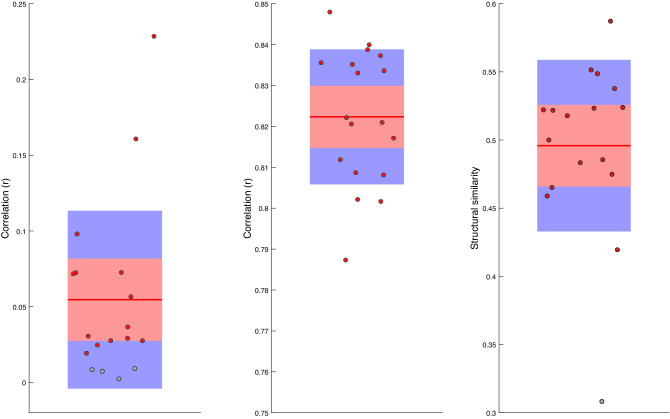
Relationships between music played to the participants and music reconstructed via our fMRI-informed EEG source analysis approach. Correlation coefficients are shown in the time domain (left) and frequency domain (centre). The right figure shows structural similarity measures between the time-frequency spectra of the original and reconstructed music. Significant measures ($p~<~0.05$) are highlighted in red/bold.

In order to quantify these similarities across all trials and participants we measured the Pearson’s correlation coefficients between the original and reconstructed music pieces in the time and frequency domains for each trial within each participant’s dataset. We also measure the similarity of the time–frequency spectra of the original and recovered music via the structural similarity index^38^. The resulting correlation coefficients and structural similarity indexes are averaged over all trials for each participant. The results, individually in the time and frequency domains, and collectively from the time-frequency spectra, along with the corresponding results of bootstrapping statistical significance testing are illustrated in Fig. 5.

It may be seen that, for the majority of participants, there are statistically significant correlations between the original and reconstructed music in both the time and frequency domains. There are also significant structural similarity index values for the time–frequency spectra of the original and recovered music. This demonstrates that, for the majority of the participants, our decoding model is able to reconstruct a significant portion of the music played to participants across different temporal and spectral locations.

### Classification of music pieces

We used the reconstructed music to attempt to identify which piece of music each of our participants was listening to within each trial. For each participant, the music reconstructed via our decoding model from each trial is compared to the the original music played to that participant in the same trial. Specifically, for a single trial, we measure the similarity between the original music and the decoded music for the trial. This similarity is ranked against the similarity between the decoded music in the trial and the original music played to that participant in all the other trials in order to measure the rank accuracy of the music decoding process for that trial.

The mean rank accuracies over all trials for each of the participants are listed in Table 2 along with the results of applying statistical significance testing to measure the statistical significance of the rank accuracies from each participant.

**Table 2:** Rank accuracies for identifying pieces of music played to participants in our joint EEG-fMRI experiments.

Participant	Rank accuracy	p
1	0.865	$<~0.01$
2	0.806	$<~0.01$
3	0.671	$<~0.05$
4	0.665	$<~0.05$
5	0.576	
6	0.928	$<~0.01$
7	0.745	$<~0.01$
8	0.767	$<~0.01$
9	0.632	
10	0.556	
11	0.567	
12	0.750	$<~0.01$
13	0.715	$<~0.01$
14	0.713	$<~0.01$
15	0.554	
16	0.842	$<~0.01$
17	0.676	$<~0.05$
18	0.898	$<~0.01$
Mean	0.718	–

It can be seen that, for the majority of our participants (13 of our 18 participants), statistically significant rank accuracies can be achieved ($p~<~0.05$). A further subset of 10 participants have mean rank accuracies that are highly statistically significant ($p~<~0.01$). This demonstrates that our proposed method for music decoding is able to reconstruct enough of the music from the EEG to allow identification of the music pieces the participants were listening to.

Music tempo is known to effect the EEG^39–42^. Therefore, we measure the effect of the tempo of our music stimuli on the performance of our decoder by measuring whether there is any correlation between the performance of our decoder and either the tempo of our music stimuli or the difference in the tempo of our music stimuli within individual trials from the distribution of tempos across all trials. In both cases we do not find significant correlations between the performance of our decoder and the tempo of the music or the distribution of music tempos across trials. This evidence, combined with our evidence described above, suggests our decoder is not simply decoding the tempo of the music but is also decoding more meaningful acoustic information, across different temporal and spectral locations, from the EEG.

### EEG only analysis

To verify that our classification approach is not influenced by noise contamination that is specific to our combined EEG-fMRI recording modality, or our use of shielded headphones to deliver our music stimuli, we repeated our analysis with a separate set of EEG data. This EEG dataset was recorded from an independent cohort of 19 participants during an EEG only music listening experiment in which speakers were placed over 1m away from participants and used to play similar types of music stimuli.

We applied the same neural decoding model to attempt to reconstruct music from the EEG recordings made from this cohort of participants. However, to compensate for the lack of fMRI data from these participants we used the averaged dipole locations over all participants from our fMRI-EEG dataset for our decoding model. We also used an averaged head model taken from all participants from our EEG-fMRI dataset.

Visual inspection of our decoded music again reveals considerable visual similarities between our original and reconstructed music. To quantify these similarities we again measure Pearson correlation coefficient’s and the structural similarity index between our original music and the music we reconstructed via our decoding model in both the time and frequency domains and from the time-frequency spectra.

We find significant ($p~<~0.05$) correlations in the time domain in 16 of our participants with a mean correlation of $r~=~0.043$). In the frequency domain we find significant ($p~<~0.05$) correlations between the power spectra of the original music and reconstructed music for all of our participants, with a mean correlation of $r~=~0.844$. We also find significant ($p~<~0.05$) structural similarity indices between the time–frequency spectra of the original music and the time-frequency spectra of the decoded music in 18 of our participants. Specifically, we find a mean structural similarity of $s~=~0.496$, indicating that, once again, our decoder is able to reconstruct aspects of the acoustic signal across multiple time and frequency locations.

To further verify that our music decoding approach was performing better than random chance at reconstructing the music participants heard in our EEG-only dataset we again repeated the classification approach first attempted with the EEG-fMRI dataset (see above). The resulting rank classification accuracies for each participant are listed in Table 3.

**Table 3:** Rank accuracies for identifying pieces of music played to participants in our EEG experiments.

Participant	Rank accuracy	p
1	0.458	
2	0.694	$<~0.01$
3	0.618	$<~0.01$
4	0.562	
5	0.528	
6	0.576	$<~0.05$
7	0.590	$<~0.05$
8	0.646	$<~0.01$
9	0.643	$<~0.01$
10	0.618	$<~0.01$
11	0.556	
12	0.604	$<~0.05$
13	0.611	$<~0.01$
14	0.646	$<~0.01$
15	0.562	
16	0.581	
17	0.465	
18	0.722	$<~0.01$
19	0.562	
Mean	0.592	–

It can be seen from this table that statistically significant ($p~<~0.05$) rank accuracies are achieved for 11 of the 19 participants in our dataset and highly significant ($p~<~0.01$) rank accuracies are achieved for 8 of the participants. This demonstrates that neural decoding of the music heard by participants is also possible on this separate EEG only dataset, albeit with a lower accuracy than achieved with the EEG-fMRI dataset. In other words, we are able to identify the music participants listened to from EEG alone using our neural decoding model, even when participant-specific fMRI data is not available to aid the source localisation step.

## Discussion

Neural decoding models provide a way to recover the original stimuli an individual is focusing on from their brain activity^1^. Decoding models have been constructed to decode numerous types of stimuli and mental states from the brain. Examples include, visual image decoding^9^, speech decoding^18,19^, and movement decoding^43^. Many previous efforts to decode complex acoustic stimuli such as music or speech have relied on neural imaging techniques that are either invasive (such as ECoG) or require detailed high spatial resolution views of sub-cortical brain regions (such as fMRI). Recent efforts have included the use of some non-invasive modalities, such as EEG, for acoustic neural decoding, with some success. However, to date the combination of EEG and fMRI has not been explored for acoustic neural decoding.

Indeed, previous research using either fMRI or ECoG has allowed decoding of heard speech, still images, movies, and even music heard by participants^20^. However, until now decoding of such complex stimuli via non-invasive, scalp based measures of neural activity such as EEG has only been demonstrated in some studies^22,23,28,30^ and there remains a need to explore how to develop effective neural decoding models that are able to decode complex information from multi-modal combinations of fMRI and EEG.

Decoding of complex information in areas such as the semantic, visual, or acoustic information domains has numerous applications. Furthermore, being able to decode such rich information sources from non-invasive, portable, and relatively cheap neuroimaging modalities such as EEG provides the potential for many exciting applications, including understanding neural processing of complex stimuli during day to day activities and developing assistive technologies such as BCI.

Indeed, one of the core goals of BCI systems is to provide fast and accurate decoding of mental states for translation into control actions. To this end neural decoders that can decode complex information, such as information in the acoustic domain, from the brain have the potential to provide improved communication quality to BCI users.

BCIs attempt to provide direct communication from the brain that works without movement and have the potential to greatly aid individuals with severe communication disabilities^17,44^. The majority of BCIs developed to date provide their users with small discrete sets of options. Brain activities such as event related potentials^45^ or conscious modulation of brain-rhythms (such as the event related de-synchronisation via imagined movement^46^) are then typically used to select between these options^47,48^.

BCIs based on decoding information in the acoustic domain, such as speech production or imagined sounds, have been proposed elsewhere^19^. However, to date, the majority of the successes achieved in this domain have made use of invasive brain imaging technologies such as electrocortiography (ECoG)^19,49–51^, which is impractical for day-to-day communication purposes for the majority of BCI users. Neural decoders that work, in the acoustic information domain, with non-invasive neural technologies, such as EEG, are, therefore, an important goal in BCI research.

We provide a demonstration of a successful attempt to build a neural decoding model in the acoustic domain for fMRI-informed EEG. Specifically, we build a decoding model that is able to reconstruct the music a participant was listening to from their EEG. Crucially, although our analysis uses an fMRI-informed EEG source analysis approach, the fMRI is only used to provide spatial localisation for EEG source analysis. This suggests that a similar decoding model could work with only EEG signals. Indeed, we demonstrate that this is in fact possible with a separate EEG only dataset.

Music is a complex acoustic signal that shares many similarities with natural language. It typically has a complex temporal profile and its own grammatical rules that share many similarities with human speech. Indeed, music has often been described as a language of emotional communication that can transcend differences in spoken language, culture, and background^52^. Furthermore, music is also a core part of the human experience for many people. It entertains us, helps with social bonding, and can enable to us to form and express a sense of identity^53^. Therefore, music decoding is a valid step to explore how acoustic information may be decoded from non-invasive recordings of brain activity.

Music, like other acoustic languages, is processed through the auditory pathway. This is a network of brain regions that carry auditory messages from the ears into and through the brain. There are two types of auditory pathway: the primary pathway, which connects to the auditory cortex and carries acoustic information, and the secondary pathway, which involves affective responses to music^36^.

The primary pathway passes auditory messages from the cochlea through the cochlear nuclei, superior olive, and inferior colliculus, to the specific thalamus, and finally on to the auditory cortex^36^. The secondary pathway passes messages from the cochlear nuclei through the reticular formation, to the thalamus and then on to the affective response network^54^, which is involved in affective responses to music and includes the limbic cortex, hypothalamus, and other parts of the cortex^36^. Our fMRI analysis results find significant differences in brain activity in parts of the brain involved in both these pathways. However, the majority of the dipoles identified for use in our fMRI-informed EEG source analysis are located in parts of the brain residing in the primary auditory pathway.

Specifically, our fMRI analysis identifies regions within the left and right transverse temporal gyrus, the left hippocampus, the cerebellum, and the left and right parahippocapal gyrus as responding to music listening by participants. These regions of the brain are well known be involved in auditory processing and music listening, so these results are not surprising. However, it is interesting to note that the specific loci of activity vary significantly between participants (this may be seen by inspecting the cloud of dipole locations in Fig. 2). This suggests that the brain regions that are most involved in processing musical stimuli vary from person to person and could help to explain why the accuracies observed in our EEG-only dataset (which uses group average dipole locations in the source analysis stage) are lower than the accuracies observed in our EEG-fMRI dataset.

Efforts to understand how our brains process music are closely related to efforts to understand how our brains process language and encode information^55,56^. Indeed there are many common brain areas that are involved in both the processing of music and other linguistic and semantic features. For example, the primary motor cortex is involved in processing music^57^ as well as differentially responding to the semantic category of specific concepts during language processing^58^.

Therefore, the identification of a decoding model that is able to use EEG to partially reconstruct the pieces of music to which an individual is listening suggests a similar approach may also allow recovery of the speech a participant is listening to. Going one step further, it is also reasonable to hypothesis that recovery of internally imagined speech via EEG—a long term goal of brain-computer interfacing—may be feasible.

Nonetheless, music decoding from the EEG is still limited by the inherent limitations of the EEG. These limitations include the lower spatial resolution afforded by this modality and the greater susceptibility of the EEG to artifacts. We have attempted to overcome these limitations in our analysis through careful use of source analysis, combined with artifact removal methods. However, our final decoding accuracies are still lower than when similar decoding models are attempted with fMRI voxel intensity values. For example, a study by Hoefle and colleagues reported a mean music decoding performance of 76% when using fMRI^20^, which compares favourably to our mean decoding performances of 71.8% and 59.2%.

Nonetheless, our results clearly demonstrate that fMRI-informed EEG analysis does enable acoustic decoding, a finding that has numerous exciting possible applications. In our future work we will investigate decoding models for heard and imagined speech from the EEG. We will also investigate the effect of different deep network architectures on our models performance.

## Methods

### Experiment

We use a joint EEG-fMRI dataset originally recorded with the intent to explore the effects of music on the emotions of the listener. The full details of the experiments are described elsewhere in Ref.^59^ and the data is publicly available^60,61^. We briefly summarise the key details of the dataset below.

#### Outline

Participants were asked to listen to two different sets of music. The first set comprised a collection of generated pieces of piano music, which had been generated to target specific affective states and was pre-calibrated to ensure they could induce the targeted affects in their listeners. The second set of music was a set of pre-existing classical piano music pieces, which were chosen for their ability to induce a wide range of different affects.

In this study we only make use of the neural data recorded during the generated music listening task. As detailed below, participants listened to a series of pieces of music over different types of trials. In some trials participants were asked to continuously report their emotions, while in others they were asked to just listen to the music.

#### Participants

A total of 21 healthy adults participated in the study. All participants were aged between 20 and 30 years old and were right-handed with normal or corrected to normal vision and normal hearing. All participants were screened to ensure they could safely participate in a joint EEG-fMRI study. Ten of the participants were female. All participants received £20.00 (GBP) for their participation.

#### Ethics

Ethical permission was granted for the study by the University of Reading research ethics committee, where the study was conducted. All experimental protocols and methods were carried out in accordance with relevant ethical guidelines. Informed consent was obtained from all participants.

#### fMRI

Functional magnetic resonance imaging (fMRI) was recorded using a 3 Tesla Siemans Magnetom Trio scanner with Syngo software (version MR B17) and a 37-channel head coil. The scanning sequence used comprised a gradient echo planar localizer sequence followed by an anatomical scan (field of view: 256 $\times$ 256 $\times$ 176 voxels, TR = 2020 ms, TE = 2.9 ms, dimensions of voxels = 0.9766 $\times$ 0.9766 $\times$ 1 mm, flip angle = 9°). This was then followed by a set of gradient echo planar functional sequences (TR = 2000 ms, echo time = 30 ms, field of view = 64 $\times$ 64 $\times$ 37 voxels, voxel dimensions = 3 $\times$ 3 $\times$ 3.75 mm, flip angle = 90°). The final sequence, applied after the music listening part of the experiment was completed, was another gradient echo planar sequence.

#### EEG

EEG was recorded via an MRI-compatible BrainAmp MR and BrainCap MR EEG system (BrainProducts Inc., Germany). EEG was recorded from 32 channels (31 channels for EEG and 1 channel for electrocardiogram) at a sample rate of 5000 Hz without filtering and with an amplitude resolution of 0.5 $\upmu$V. A reference channel was placed at position FCz on the international 10/20 system for electrode placement and all impedance’s on all channels were kept below 15 k$\Omega$ throughout the experiment.

Co-registration of the timing of the EEG and fMRI recordings was achieved by a combination of the BrainVision recording software (BrainProducts, Germany), which recorded trigger signals from the MRI scanner, and custom written stimuli presentation software written in Matlab (Mathworks, USA) with Psychtoolbox^62^.

#### Stimuli

The music played to the participants was generated with the intention of inducing a wide range of different affective states. In total 36 different musical pieces were generated to target 9 different affective states (combinations of high, neutral, and low valence and arousal).

Each piece of music was 40 s long and was generated by an affectively driven algorithm composition system that was based on an artificial neural network^63^ that had been previously validated on an independent pool of participants^64^. The resulting music was a piece of mono-phonic piano music as played by a single player.

#### Tasks

The experiment was divided into a series of individual tasks for the participants to complete. These tasks fell into three different types:

1. *Music only trials* In these trials participants were asked to just listen to a piece of music.
2. *Music reporting trials* In these trials participants were asked to listen to a piece of music and, as they listened, to continuously report their current felt emotions on the valence-arousal circumplex^65^ via the FEELTRACE interface^66^.
3. *Reporting only trials* These trials were used to control for effects of motor control of the FEELTRACE interface. Participants were shown, on screen, a recording of a previous report they had made with FEELTRACE and were asked to reproduce their recorded movements as accurately as they could. No music was played during these trials.

Within each trial participants were first presented with a fixation cross, which was shown on screen for 1–3 s with a random, uniformly drawn, duration. The task then took 40 s to complete and was then followed by a short 0.5 s break.

All sound was presented to participants via MRI-compatible headphones (NordicNeurolab, Norway). Participants also wore ear-plugs to protect their hearing and the volume levels of the music were adjusted to a comfortable level for each participant before the start of the experiment.

The trials were presented in a pseudo-random order and were split over 3 runs, each of which was approximately 10 min long. A 1-min break was given between each pair of runs and each run contained 12 trials in total.

### Pre-processing

Both the EEG and the fMRI signals were pre-processed to remove artefacts and allow for further analysis.

#### fMRI

The fMRI data was pre-processed using SPM12 software^67^ running in Matlab 2018a.

Slice time correction was applied first, using the first slice of each run as the reference image. This was followed by removal of movement related artefacts from the images via a process of realignment and un-warping using the approach originally proposed by Friston et al.^67^. The field maps recorded during the scan sequences were used to correct for image warping effects and remove movement artefacts. A 4 mm separation was used with a Gaussian smoothing kernel of 5 mm. A 2nd degree spline interpolation was then used for realignment and a 4th degree spline interpolation for un-warping the images.

We then co-registered the functional scans against the high-resolution anatomical scan for each participant before normalising the functional scans to the high-resolution anatomical scan.

Finally, the functional scans were smoothed with a 7 mm Gaussian smoothing kernel and a 4th degree spline interpolation function.

#### EEG

The fMRI scanning process induces considerable artefacts in the EEG. To remove these mechanistic artefacts the Average Artefact Subtraction (AAS) algorithm was used^68^. The version of AAS implemented in the Vision Analyser software (BrainProducts, Germany) was used. The cleaned EEG was then visually checked to confirm that all the scanner artefacts had been removed.

Physiological artefacts were then manually removed from the signals. The EEG was first decomposed into statistically independent components (ICs) by application of second order blind identification (SOBI)^69^ a variant of independent component analysis that identifies a de-mixing matrix that maximises the statistical independence of the second order derivatives of the signals.

Each resulting IC was then manually inspected in the time, frequency, and spatial domains by a researcher with 10+ years experience in EEG artifact removal (author ID). Components that were judged to contain artefacts (physiological or otherwise) were manually removed before reconstruction of the cleaned EEG. A final visual inspection of the cleaned EEG was performed to confirm that the resulting signals are free from all types of artifact.

### fMRI analysis

The fMRI dataset was used to identify voxels with activity that significantly differs between music listening (trials in which participants listen to music only and trials in which participants both listen to music and report their current emotions) and non-music listening trials (trials in which participants only use the FEELTRACE interface without hearing music).

Specifically, a general linear model was constructed for each participant and used to identify voxels that significantly differ (T-contrast) between these two conditions. Family-wise error rate was used to correct for multiple comparisons (correct p < 0.05). The resulting clusters of voxels were used to identify brain regions which exhibit activity that significantly co-varies with whether the participants were listening to music or not.

### Source localisation

An fMRI-informed EEG source localisation approach was used to extract EEG features that are most likely to be informative for reconstruction of the music participants listened to from their neural data. To this end we first built a high resolution accurate conductivity model of the head. We then used a beam-former source reconstruction method, implemented in Fieldtrip^70^, to estimate the activity at a set of individual source locations in the brain. These source locations were chosen based on the fMRI analysis results on a per participant basis.

**Figure 6:**
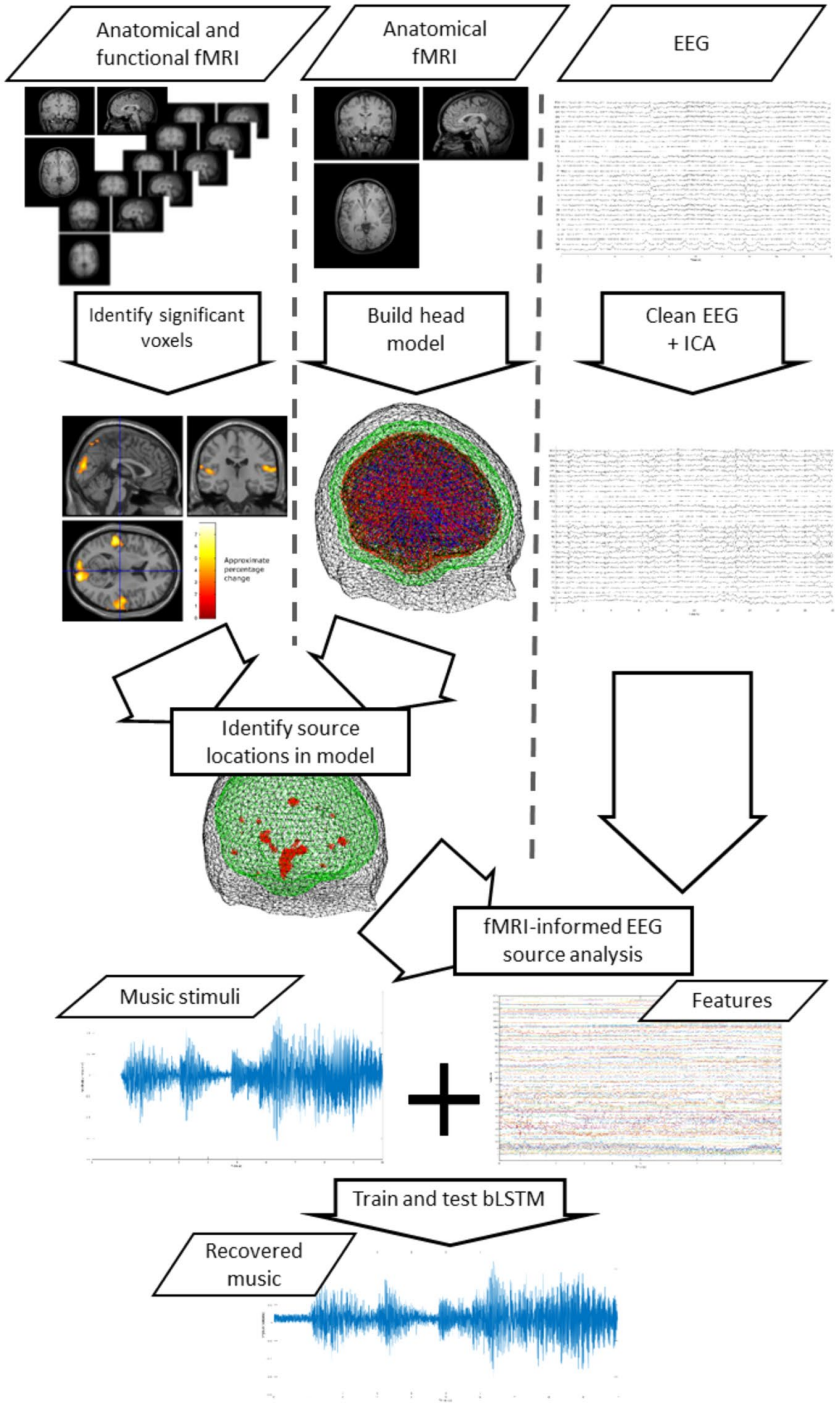
Analysis pipeline illustration. Anatomical MRI is used to construct head models, while fMRI is used to identify voxels that differ between music and no music conditions. EEG is decomposed via ICA and fMRI-informed source analysis is used to characterise activity at fMRI-identified locations. The resulting feature set is used to train a biLSTM to recover the music a participant listened to. A cross-fold train and validation scheme is used for each participant.

The entire process is illustrated in Fig. 6.

#### Model construction

A detailed head model was constructed for each participant to model conductivity within the head from each participant’s individual anatomical MRI scan. Fieldtrip was used to construct this model^70^.

The anatomical scan from each participant was first manually labelled to identify the positions of the nasion and the left and right pre-auricular points. The scan was then segmented into gray matter, white matter, cerebral spinal fluid, skull, and scalp tissue using the Fieldtrip toolbox^70^. Each segmentation was then used to construct a 3-dimensional mesh model out of sets of vertices (3000 vertices for the gray matter and the cerebral spinal fluid, 2000 vertices for each of the other segments). These mesh models were then used to create a conductivity model of the head via the finite element method^71,72^. We specified the conductivity of each layer using the following standardised values: gray matter $=0.33$ S/m, white matter $=0.14$ S/m, cerebral spinal fluid $=1.79$ S/m, skull $=0.01$ S/m, and scalp $=0.43$ S/m. These values were chosen based on recommendations in^71,73,74^.

The EEG channel locations were then manually fitted to the model by a process of successive rotations, translations, and visual inspection. Finally, a lead-field model of the dipole locations inside the conductivity model was computed from a grid of 1.5 $\times$ 1.5 $\times$ 1.5 cm voxels.

#### Source estimation

Source estimation was achieved by using the conductivity head model and the eLoreta source reconstruction method^75,76^ to estimate the electro-physiological activity at specific voxel locations within the head model. Specifically, voxel locations in the model were chosen based on the results of analysis of the fMRI datasets (see the “fMRI analysis” section).

From the set of voxels that were identified, via the GLM, as containing activity that significantly differs between the music and no music conditions a sub-set of voxel cluster centres were identified as follows.

1. Begin with an empty set of voxel cluster locations *V* and a set of candidate voxels *C*, which contain all the voxels identified via our GLM-based fMRI analysis as significantly differing between the music and no music trials.
2. Identify the voxel with the largest T-value (i.e. the voxel that has the largest difference in variance between the music and no music conditions).
3. Measure the Euclidean distance between the spatial location of this voxel in the head and all voxels currently in the set *V*. If the smallest distance is greater than our minimum distance *m* add it to the set *V*.
4. Remove the candidate voxel from the set *C*.
5. Repeat steps 2–4 until the set *V* contains $n_l$ voxels.

This process ensures that we select a sub-set of voxel locations that differentiate the music and no music trials, while ensuring this set of voxels are spatially distinct from one another. This results in a set of $n_l$ voxel locations that characterise the distributed network of brain regions involved in music listening. In our implementation we set the minimum distance *m* = 3 cm and $n_l$ = 4 voxel locations.

### Feature set construction

To extract a set of features from the EEG to use for reconstructing the music played to participants we first use independent component analysis (ICA) to separate the EEG into statistically independent components. Each independent component is then projected back to the EEG electrodes by multiplying the component by the inverse of the de-mixing matrix identified by the ICA algorithm. This gives an estimate of the EEG signals on each channel if only that independent component were present.

This IC projection is then used, along with the pre-calculated head model for the participant, to estimate the source activity at each of the $n_l$ = 4 locations identified by our source estimation algorithm (see “Source estimation” section). This results in a matrix of 4 $\times N_s$ sources for each IC projection, where $N_s$ denotes the total number of samples in the recorded EEG signal set. These matrices are generated for each IC projection and concatenated together to form a feature matrix of dimensions $(4 \times M) \times N_s$, where *M* denotes the number of EEG channels (31 in our experiment). Thus, our final feature vector is a matrix of EEG source projections of dimensions $124 \times N_s$.

### Music prediction

**Figure 7:**
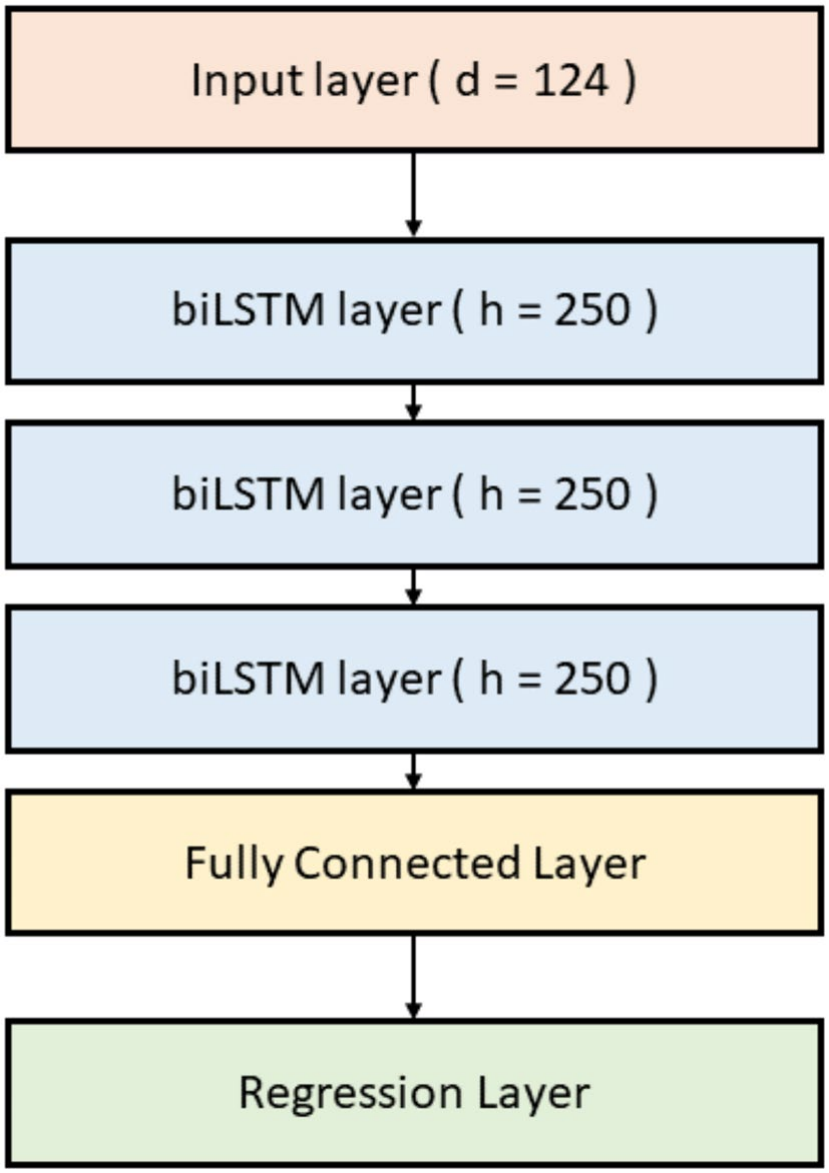
Architecture of the biLSTM used to attempt to recover heard music from our fMRI-informed EEG source analysis.

Reconstruction of the music participants heard from fMRI-informed EEG sources is attempted via a deep neural network. Specifically, a stacked 4-layer bi-directional long short-term memory (biLSTM) network is constructed. The first layer is a sequence input layer with the same number of inputs as features (124). Four biLSTM layers are then stacked, each with 250 hidden units. A 1-layer fully connected layer is then added to the stack, followed by a regression layer. The architecture of the biLSTM network is illustrated in Fig. 7.

The music played to each participant is down-sampled to the same sample rate as the EEG (1000 Hz). Both the music and the feature vector (see the “Feature set construction” section) are then further down-sampled by a factor of 10 from 1000 to 100 Hz.

The network is trained and tested to predict this music from the EEG sources within a 3 $\times$ 3 cross-fold train and test scheme. Specifically, each run of the 3 runs from the experiment is used once as the test set in each fold. The training and testing data comprise the time series of all EEG sample points and music samples from all time points when the participants listened to music (trial types 1 and 2, see the experiment description above) within each run.

### Statistical analysis

We evaluate the performance of our decoding model in several ways.

First, we compare the time series of the reconstructed music with the original music played to the participants via visual inspection and via a correlation analysis in both the time and frequency domains. Specifically, the Pearson’s correlation coefficient between the original and reconstructed music (downsampled to 100 Hz), in the time domain is measured. We then compare the power spectra of the original and reconstructed music via Pearson’s correlation coefficient. We also measure the structural similarity^38^ of the time–frequency spectrograms between the original and reconstructed music.

For each of these indices of similarity between the original and reconstructed music we measure the statistical significance via a bootstrapping approach. We first generate sets of reconstructed music under the null hypothesis that the reconstructed music is not related to the original music stimuli by shuffling the order of the reconstructed music trials. We repeat this 4000 times for each similarity measure (correlation coefficients and structural similarity) and measure the similarity between the original music and the shuffled reconstructed music in each case in order to generate null distributions. The probability that the measured similarity between the original music and the un-shuffled reconstructed music is drawn from this null distribution is then measured in order to estimate the statistical significance of the similarity measures.

Second, we use the reconstructed music to attempt to identify which piece of music a participant was listening to within each trial. If the decoding model is able to reconstruct a reasonable approximation of the original music then it should be possible to use this reconstructed music to identify which specific piece of music a participant was listening to in each trial.

Specifically, we first z-score the decoded and original music time series in order to remove any differences in amplitude scaling. We then band-pass filter both signals in the range 0.035 Hz to 4.75 Hz. These parameters were chosen to preserve the visually apparent similarities in the amplitude envelopes of the original and decoded music, which were observed upon visually inspecting a subset of the data (participants 1 and 2).

We then segmented the signals into individual trials as defined by the original experiment. Specifically, each trial is 40 s long and comprises a single piece of music. For a given trial the structural similarity is measured between the spectra of the original music played to the participant in that trial and the spectra of the reconstructed music. The structural similarity is then also measured between the time-frequency spectra of the reconstructed music for that same trial and the time–frequency spectra of the original music played to the participant in all the other trials in which the participant heard a different piece of music. Specifically, we measure

1 $$\begin{aligned} C_{k,k} = \text{ ssim }( R_k, M_k ), \end{aligned}$$

and

2 $$\begin{aligned} C_{k,i} = \text{ ssim }( R_k, M_i )~~~~~~~~\forall \ i \in A, \end{aligned}$$

where $R_k$ denotes the time–frequency spectrogram of the reconstructed music for trial *k*, $M_i$ denotes the time–frequency spectrogram of the original music played to the participant in trial *i*, and $\text{ ssim }$ indicates the use of the structural similarity measure. For a given trial *k* the value of $C_{k,i}$ is measured for all trials in the set *A* ($i \in A$), where *A* is defined as

3 $$\begin{aligned} A = \{1,...N_t\} \setminus k, \end{aligned}$$

and denotes the complement of set of all trials $1, ..., N_t$ (where $N_t$ denotes the number of trials) and the trial, *k*, for which we reconstructed the music played to the participant via our decoding model.

We then order the set of structural similarity measures $C = {C_{k,i}}~\forall i \in 1,...,N_t$ and identify the position of $C_{k,k}$ in this ordered list in order to measure the rank accuracy of trial *k*. Rank accuracy measures the normalised position of $C_{k,k}$ in the list and is equal to 0.5 under the null hypothesis that the music cannot be identified. In other words rank accuracy measures the ability of our decoder to correctly decode our music by measuring how similar the decoded and original music are to one another compared to the similarity between the decoded music and all other possible pieces of music. Finally, we measure the statistical significance of our rank accuracy via the method described by Ref.^77^.

### Effect of tempo

A number of studies have reported significant effects of music tempo on the EEG^39–42^. Therefore, we investigate whether the tempo of the music played to participants significantly effects the performance of our decoding model.

Specifically, we estimate the range of tempos within each 40 s long piece of music stimuli and the corresponding mean tempo. We then test whether the mean tempo of the music significantly effects the performance of our decoding model by measuring the Pearson’s correlation coefficient between the mean tempo of the music played to the participant within each trial and the corresponding rank accuracy measure of the decoders performance for that same trial. Additionally, we also measure the likelihood that the mean tempo for the music within a single trial was drawn from the distribution of mean tempos over all trials. This allows us to estimate whether the tempo of the music within a trial is ‘typical’ or less ‘typical’. We measure the correlation between this measure of the typicality of the tempo of the music and the performance of the decoder on this trial to identify whether trials with unusual tempos (faster or slower than usual) are classified more (or less) accurately.

In both cases we hypothesise that if our decoder is predominately making use of the tempo of the music there will be significant correlations between the decoder’s performance and either the tempo of the music or the likelihood (typicality) of the tempo of the music.

### Confound consideration

The use of headphones to play music to participants presents one potential confounding factor in our analysis. Although the headphones we used were electromagnetically shielded in a way that is suitable for use within an fMRI scanning environment there is a possibility that their proximity to the EEG electrodes lead to some induced noise in the recorded EEG signals. This noise could either be electromagnetic noise from the electrical operation of the headphones of vibrotactile from the vibration of the headphones.

We expect that if this is the case the noise removal applied to the EEG should remove this noise. Indeed, our visual inspection of our cleaned EEG signals did not reveal any apparent induced noise. However, we cannot discount the possibility that some residual noise from the headphones (either electromagnetic or vibrotactile in nature) remains in the EEG signal and that this is used as part of the decoding process.

The only way to verify that this was not the case is to attempt to repeat the experiment without the use of headphones. Therefore, we make use of another dataset recorded by our team^78^ using conventional speakers placed over 1 m away from participants to play similar pieces of music. This dataset contains just EEG recorded from participants while they listened to similar sets of synthetic music stimuli in a separate experiment. As this dataset only contains EEG data participant specific fMRI-informed source analysis is not possible. Instead, we use the averaged fMRI results from all our participants in our EEG-fMRI experiments to provide an averaged head model and averaged source dipole locations for the fMRI-informed source analysis step in our decoding pipeline.

We first detail this dataset and then go on to describe how we adapted our analysis pipeline to attempt to decode music played to participants in this experiment.

#### Dataset

Our EEG only dataset was originally recorded as part of a set of experiments to develop an online brain-computer music interface (BCMI). These experiments, their results, and the way the dataset is recorded are described in detail in Ref.^78^. We also describe the key details here.

A cohort of 20 healthy adults participated in our experiments. EEG was recorded from each participant via 32 EEG electrodes positioned according to the international 10/20 system for electrode placement at a sample rate of 1000 Hz.

Participants were invited to participate in multiple sessions to first calibrate, then train, and finally to test the BCMI. For our purposes in this present study we only use the EEG data recorded from participants during the calibration session.

In the calibration session a series of synthetic music clips were played to participants. Each clip was 20 s long and contained pre-generated piano music. The music was generated by the same process used for our EEG-fMRI experiments (see the “Stimuli” section). A total of 90 unique synthetic music clips were played to the participants in random order. Each clip was generated for the purpose of the experiment (ensuring the participants had never heard the clip before) and targeted a specific affective state. Participants were instructed to report their current felt affect as they listened to the music using the FEELTRACE interface in a similar way to the joint EEG-fMRI experiments described above.

Details of the dataset and accompanying stimuli are described in Refs.^61,78^. The data is also published in Ref.^79^.

#### Ethics

Ethical permission for recording this second dataset was also granted by the University of Reading research ethics committee, where the study was originally conducted. All experimental protocols and methods were carried out in accordance with relevant ethical guidelines. Informed consent was obtained from all participants.

#### Analysis

Our decoding model is modified slightly to attempt to reconstruct the music played to participants in the EEG-only experiments. Specifically, we use the mean average of the fMRI results from our cohort of participants in our joint EEG-fMRI dataset to identify the set of voxels for use in our fMRI informed EEG analysis. Furthermore, our head model used in the fMRI-informed EEG source analysis step in our decoding model is constructed from an averaged MRI anatomical scan provided within SPM12^67^.

All other stages of our decoding model and analysis pipeline—including EEG source localisation, biLSTM network structure, and statistical analysis—are the same.

## Data Availability

All data used in this study are publicly available. The fMRI-EEG dataset is available at Ref.^60^, while the EEG-only dataset is available at Ref.^79^.

## Code availability

The software used in this study is Matlab 2018a, the Statistical Parametric Mapping toolbox (SPM12^67^), and the Vision Analyser software (BrainProducts, Germany), all of which are publicly available.
